# The impact of *Helicobacter pylori* infection and eradication therapies on gut microbiota: a systematic review of microbial dysbiosis and its implications in gastric carcinogenesis

**DOI:** 10.3389/fcimb.2025.1592977

**Published:** 2025-07-07

**Authors:** Alia Albush, Fayez Yassine, Hassan Abbas, Aya Hanna, Esber Saba, Melhem Bilen

**Affiliations:** ^1^ Department of Experimental Pathology, Immunology and Microbiology, Faculty of Medicine, American University of Beirut, Beirut, Lebanon; ^2^ Center for Infectious Diseases Research, American University of Beirut, Beirut, Lebanon; ^3^ World Health Organization (WHO) Collaborating Center for Reference and Research on Bacterial Pathogens, Beirut, Lebanon

**Keywords:** *H. pylori*, eradication therapies, gut microbiota, gastric cancer, probiotics

## Abstract

**Background:**

*Helicobacter pylori* is a globally prevalent bacterium associated with several gastrointestinal diseases, including peptic ulcers and gastric cancer. Growing interest has emerged in understanding how *H. pylori* affects gut microbiota and whether eradication therapies impact microbial balance, potentially influencing disease outcomes, including cancer progression.

**Methods:**

A systematic review was conducted across PubMed, Scopus, and Web of Science databases using predefined keywords and Medical Subject Headings (MeSH) terms. Quality assessment was performed using the MINORS and Jadad scales.

**Results:**

A total of 45 studies met the inclusion criteria, which evaluated microbial changes in *H. pylori* -infected individuals before and after eradication therapies. *H. pylori* infection resulted in significant alterations in gut and gastric microbiota, with a notable increase in inflammation-associated bacteria, such as *Proteobacteria* and *Streptococcus*. In gastric cancer patients, microbial diversity was reduced, with decreased levels of *Bifidobacterium* and *Actinobacteria*, and increased levels of *Prevotella* and *Dialister*, both associated with pro-inflammatory environments. Eradication therapies generally worsened dysbiosis initially, but probiotic supplementation promoted faster recovery of beneficial bacteria, improving microbial balance and reducing cancer-related dysbiosis.

**Conclusion:**

*H. pylori* infection disrupts the gut microbiota, with eradication therapies further altering microbial composition. The restoration of microbial diversity is improved by probiotic supplementation. Understanding the long-term impacts of these therapies on gut health is essential for refining treatment strategies, particularly in preventing *H. pylori* -associated diseases like gastric cancer.

## 1 Introduction

*Helicobacter pylori* (*H. pylori*) is a small, spiral-shaped Gram-negative bacterium that measures between 0.5 to 1 μm in width and 2 to 4 μm in length (Öztekin et al., 2021). Its discovery in the 1980s transformed the understanding of gastrointestinal diseases, particularly peptic ulcers, which were previously attributed to stress and diet (Bornschein and Pritchard, 2022). The unique ability of *H. pylori* to survive in the acidic environment of the human stomach allows it to colonize over 50% of the global population, though many individuals remain asymptomatic (Frost et al., 2019; Fong, 2020; Charitos et al., 2021). In others, however, the infection can lead to gastritis, peptic ulcer disease, or even gastric cancer (Duan and Xu, 2025).

The bacterium survives the acidity of the stomach by producing urease, an enzyme that neutralizes stomach acid by converting urea into ammonia (Cunha et al., 2021). While this adaptation aids in its colonization, it also damages the stomach lining, contributing to ulcer formation in some people (Ali and AlHussaini, 2024). Testing for *H. pylori* is essential in patients with peptic ulcer symptoms or a family history of gastric cancer, though routine screening is not advised unless there is a clinical need (Garman et al., 2024). Beyond urease, other virulence factors, such as vacuolating cytotoxin A (vacA) and cytotoxin-associated gene A (cagA protein), are linked to varying disease outcomes (Baj et al., 2020). Notably, the cagA-positive strain of *H. pylori* is associated with a higher risk of gastric cancer (Peng et al., 2024).However, the reasons why some individuals develop severe disease while others remain asymptomatic remain unclear (Alexander et al., 2021).

Recent research has focused on the gut microbiota in patients with *H. pylori* infections, particularly in light of growing evidence linking gut microbiota dysbiosis with various health conditions (Yadav and Chauhan, 2024). Dysbiosis, or the imbalance in gut microbial communities, has been associated with gastrointestinal diseases and systemic conditions such as metabolic syndrome and cardiovascular disease (Yu et al., 2021). Importantly, dysbiosis is also implicated in the development of gastric cancer, which is the third leading cause of cancer-related deaths globally (Huang et al., 2023). *H. pylori* is believed to contribute to dysbiosis by inducing chronic inflammation, altering immune responses, and disrupting the natural microbial balance of the gut (Bakhti and Latifi-Navid, 2021). This disruption weakens the defenses of the stomach and facilitates the development of pre-cancerous lesions, which may promote the growth of harmful bacteria that accelerate cancer progression (Bakhti and Latifi-Navid, 2021).

This systematic review explores the relationship between gut microbiota and *H. pylori*, focusing on the gastrointestinal changes observed with and without therapeutic intervention. Additionally, it examines the impact of *H. pylori* on gut microbiota in patients with gastric cancer, highlighting the potential role of these microbial shifts in cancer progression.

## 2 Methodology

### 2.1 Search method and databases

A comprehensive search strategy was performed by multiple reviewers across PubMed, Scopus, and Web of Science using three keyword and MeSH term lists. These keywords were linked with the OR function, and lists were combined with the AND function, while the NOT function excluded review articles (**Supplementary Data 1**). No restrictions were placed on publication dates. Duplicates were removed using Rayyan AI and verified manually (**Supplementary Data 2**). The screening process included two stages: an initial independent screening of titles and abstracts, followed by a full-text review to confirm inclusion criteria. Data extraction involved details such as title, publication year, patient numbers, country, age, sample type, treatment regimen, microbial changes in *H. pylori*-positive patients, and consensus was achieved through discussion.

### 2.2 Inclusion criteria

The systematic review included original articles (Randomized Controlled Trials, Cohort Studies, Case-Control Studies, Cross-Sectional Studies, and Longitudinal Studies). Studies examining the impact of *H. pylori* infection without treatment on the composition of the gut and gastric microbiota, the effects of various *H. pylori* eradication antibiotic regimens on gut and gastric microbiome composition, and research reporting microbiota composition analyses in *H. pylori*-positive patients with gastric cancer were also considered.

### 2.3 Exclusion criteria

Studies that are not original articles (Reviews, Meta-analyses, Editorials, Chapters, Reports), involving animal models, or involving patients with recent antibiotic use were excluded.

### 2.4 Quality assessment

The quality of the included studies was assessed using the MINORS and Jadad scales. The MINORS tool, commonly used in systematic reviews and meta-analyses, was applied for non-randomized studies, distinguishing between comparative and non-comparative designs (Slim et al., 2003). Comparative studies were scored out of 24, while non-comparative studies were scored out of 16, with studies categorized into quartiles based on their scores. The Jadad scale was used for randomized controlled trials (RCTs), evaluating randomization, blinding, and withdrawals/dropouts, with scores ranging from 0 to 5, where higher scores indicated better quality (De Cassai et al., 2023).

## 3 Results

### 3.1 Search strategy outcomes and quality evaluation

#### 3.1.1 Databases search outcomes

Two search queries identified relevant studies. Query 1 initially retrieved 1,640 articles, reduced to 808 after removing duplicates, while Query 2 retrieved 371, reduced to 208. From these, 307 articles from Query 1 and 192 from Query 2 were excluded as they were primarily reviews or animal studies. Further, 460 articles from Query 1 were removed, and 9 from Query 2 for being off topic based on title-abstract review. Ultimately, after retrieving full texts one study from Query 1 was excluded due to recent antibiotic use, and two from Query 2 were excluded for addressing gastric diseases unrelated to gastric cancer, resulting in 45 studies included in the systematic review. A PRISMA diagram summarizes this process (**Supplementary Data 3**).

#### 3.1.2 Quality assessment

All 37 non-randomized articles were evaluated using the MINORS scale, which is suitable given the observational design of the included studies. The overall average MINORS score is 16.13± 3.47, with scores ranging from 9 to 21. Comparative studies have an average score of 17.82± 1.31 (range 16-21), while non-comparative studies have an average of 10 ± 0.53 (range 9-11). Among the comparative studies, 23 are in the third quartile and 6 in the fourth, while the 8 non-comparative studies are in the third quartile (**Supplementary Table S1**). For the randomized studies, all 8 studies were evaluated using the Jadad scale. The overall average Jadad score is 3.5± 0.74, with scores ranging from 3-5 (**Supplementary Table S2**).

### 3.2 Findings of studies evaluating the gut microbiota of *Hp+* patients without treatment

In this section (**Supplementary Table S3**), 14 studies were analyzed. In total, gastric microbiota signatures were evaluated in 207 children (Age range: <14 years), 159 adolescents (Age range: 14–17 years), and 1,148 adults (Age range: >18). Various sample types were analyzed, including gastric biopsies, gastric fluid, and stool samples. The methods employed for taxonomic analysis included: 16S rRNA gene sequencing, shotgun metagenomic sequencing, and whole-genome sequencing.

In studies focusing on children and adolescents, alpha diversity was reported to increase in three studies (two stool samples, one gastric fluid) and decrease in two studies (gastric biopsies). Beta diversity remained unchanged in one study (gastric fluid), decreased in one (gastric biopsy), and distinguished between *H. pylori*-positive and -negative groups in two studies (gastric biopsy, stool). In gastric biopsy samples, *Actinobacteria, Bacteroidetes*, and Firmicutes levels decreased, while *Proteobacteria* levels increased in two studies. In gastric fluid samples, *Actinobacteria* and *Lactobacillus* levels decreased, whereas Streptococcus levels increased. In stool samples, *Actinobacteria, Bacteroidetes, Clostridium, Eubacterium* (in two studies), *Firmicutes, Lactobacillus, Prevotella* (in two studies), and *Proteobacteria* levels increased. *Bifidobacterium* levels showed mixed results, decreasing in one study and increasing in another, while *Streptococcus* levels increased in one study and remained unchanged in another (**Supplementary Table S3**).

In adult populations, alpha diversity increased in four studies (three stool, one gastric biopsy), decreased in three (two gastric biopsy, one stool), and showed no significant change in one (stool). Beta diversity differed between Hp+ and Hp- groups in five studies (three stool, two gastric biopsy). *Lactobacillus* levels decreased in one study (gastric biopsy) and showed no significant change in another (stool). *Proteobacteria* increased in two studies (stool, gastric biopsy) and decreased in one (gastric biopsy). *Prevotella* levels increased in three studies (two stool, one gastric biopsy), while *Bifidobacterium* decreased in one study (gastric biopsy) with no significant change in another (stool). *Haemophilus* increased in two studies (gastric biopsy, stool), and *Verrucomicrobia* phyla decreased in two studies (stool, gastric biopsy), with one study also reporting a decrease in *Akkermansia muciniphila* at the species level (stool), although this specie showed no significant change in another study (stool). The *Acinetobacter* genus decreased in one study, while the species *Acinetobacter baumannii* increased in another (both gastric biopsy). *Bacteroides* decreased in three studies (two stool, one gastric biopsy), and *E. coli* decreased in two studies (gastric biopsy, stool). One study found that the overall composition of the gastric microbiota in *H. pylori*-infected adults was like that of non-infected adults (gastric fluid) (**Supplementary Table S3**).

### 3.3 Findings of the studies evaluating the gut microbiota of *Hp*+ patients after different treatments

The population characteristics in the studies mainly focus on adult subjects, with sample sizes ranging from 11 to 1,214 participants (**Supplementary Table S4**). The age range of participants is typically between 3 and 80 years. The studies primarily utilize stool samples to analyze gut microbiota changes, after various therapeutic regimens for *H. pylori*. The methods used for taxonomic analysis are as follows: 81% of the studies employed 16S rRNA gene sequencing, 11% applied quantitative bacteriological culture techniques, 7% utilized shotgun metagenomic sequencing, and 3% used whole genome sequencing.

A total of 18 studies evaluated eradication therapies without probiotic supplementation, 9 of which employed triple therapy. Within 1–2 weeks of treatment initiation, these studies demonstrated variable effects on microbial diversity. Among those using triple therapy, 33.33% reported a decrease in alpha diversity, 22.22% showed significant changes in beta diversity, and 11.11% reported no significant change in beta diversity.

7 studies investigated the impact of bismuth quadruple therapy. At 1–2 weeks post-treatment, alpha diversity was reduced in 71.4% of studies, and beta diversity changes were significant in 57.1%. 3 studies assessed the effects of standard (non-bismuth) quadruple therapy. Within 1–2 weeks, alpha diversity decreased in 33.33% of studies, increased in another 33.33%, and beta diversity changes were significant in 66.66%. Levofloxacin-based quadruple therapy was evaluated in one study, which reported a reduction in alpha diversity and significant beta diversity alterations at week 2. High-dose dual therapy (HDDT) was investigated in a single study, where both alpha diversity was reduced and beta diversity significantly altered at week 2. Another study assessed vonoprazan–amoxicillin dual therapy (VA-dual therapy), which showed no significant changes in either alpha or beta diversity at 1 week. Polaprezinc-containing therapy was reported in one study, with no significant changes observed in alpha diversity at week 4.

At 2–3 months post-treatment, alpha diversity decreased in 11.1% of studies, showed no significant change in 16.6%, and had mixed outcomes in 27.7%, with reductions noted in C10, BQT, and EAML therapies at the genus level and VACT therapies. No significant change was seen with T14, VA dual therapy, HDDT, EAML at the species level, and PQT. Additionally, 5.5% of studies reported incomplete recovery, and another 5.5% showed restoration. Beta diversity showed significant changes in 5.5% of studies, recovery in 5.5%, no significant change in 11.1%, and mixed results in 22.2%. Restoration of beta diversity was observed with T14 therapy and EAML therapy at the species level, while significant changes were reported with C10, BQT, EAML at the genus level, and VAC TT therapies. In contrast, VA Dual therapy showed no significant beta diversity change.

To facilitate clearer comparison and summary of the findings across different time points, the results are organized into the following tables. The impact of *H. pylori* eradication therapies on gut microbiota composition was assessed at both mid-term (2–3 months) and long-term (1 year) follow-ups. At 2–3 months, notable changes were observed across several phyla, with frequent decreases in *Actinobacteria* and *Firmicutes*, and mixed responses in *Bacteroidetes* and *Proteobacteria* (**Table 1**). One year post-treatment, alpha and beta diversity showed partial restoration in some studies, though changes persisted in others (**Table 2**). Phylum-level analysis at the 1-year mark revealed improved recovery trends in *Firmicutes*, *Bacteroidetes*, and *Proteobacteria*, while *Verrucomicrobiota* and Actinobacteria remained variably affected (**Table 3**). The impact of H. pylori eradication therapies on gut microbiota composition was assessed at both mid-term (2–3 months) and long-term (1 year) follow-ups. At 2–3 months, notable changes were observed across several phyla, with frequent decreases in *Actinobacteria* and *Firmicutes*, and mixed responses in *Bacteroidetes* and *Proteobacteria* (**Table 1**). One year post-treatment, alpha and beta diversity showed partial restoration in some studies, though changes persisted in others (**Table 2**). Phylum-level analysis at the 1-year mark revealed improved recovery trends in *Firmicutes*, *Bacteroidetes*, and *Proteobacteria*, while *Verrucomicrobiota* and *Actinobacteria* remained variably affected (**Table 3**).

**Table 1:** Phylum-level gut microbiota changes observed 2–3 months after *H. pylori* eradication therapy.

Phylum	▲ Increase	▼ Decrease	➖ No change	✅ Restored
Fusobacteria	—	—	11.1%	5.5%
Bacteroidetes	11.1%	—	5.5%	16.6%
Firmicutes	—	11.1%	11.1%	16.6%
Proteobacteria	5.5%	11.1%	5.5%	11.1%
Actinobacteria	—	22.2%	—	16.6%
Verrucomicrobiota	—	5.5%	—	5.5%

This table illustrates the mid-term impact of antibiotic and probiotic interventions on dominant gut microbial phyla, with percentages representing the proportion of studies reporting each observed change.

**Table 2:** Long-term (1-Year) changes in alpha and beta diversity following *H. pylori* eradication therapy.

Type	✅ Restored	➖ No change
Alpha	11.1%	5.5%
Beta	11.1%	5.5%

This table presents long-term (1-year) changes in gut microbiome diversity following antibiotic and probiotic interventions, with percentages indicating the proportion of studies reporting each observed outcome.

**Table 3:** Phylum-level gut microbiota changes observed 1 year after *H. pylori* Eradication Therapy.

Phylum	▲ Increase	▼ Decrease	➖ No Change	✅ Restored	○ Almost Restored
Fusobacteria	5.5%	—	5.5%	5.5%	—
Bacteroidetes	—	5.5%	—	11.1%	—
Firmicutes	5.5%	—	—	11.1%	—
Proteobacteria	5.5%	—	—	11.1%	—
Actinobacteria	5.5%	—	—	5.5%	—
Verrucomicrobiota	—	—	—	5.5%	5.5%

This table summarizes microbiome shifts at the phylum level observed over the course of one year. Each percentage represents the proportion of studies (not individual participants) reporting a particular type of change.

Among the 18 included studies, 5 conducted comparative analyses between different *H. pylori* eradication therapies. These studies consistently showed that bismuth-based quadruple therapies (BQ10 and BQT) were associated with greater reductions in alpha diversity compared to other regimens such as T14, EAML14, and PQT. Beta diversity significantly distinguished treatment groups in all five studies, indicating distinct shifts in microbial community structure. In particular, BQT was linked to slower microbial recovery and greater dysbiosis compared to HDDT and PQT. Taxonomic analyses revealed increased *Proteobacteria* and decreased *Firmicutes* and *Bacteroidetes* shortly after treatment, with partial restoration over time. Species-level changes, such as elevated *K.pneumoniae* and *Enterococcus* spp., were more pronounced in the BQT group. Additionally, certain taxa like *Parasutterella*, *Ruminococcus*, and *Anaerostipes* were differentially abundant across treatment arms, highlighting therapy-specific microbial impacts.

It is worth noting that while all the included studies assessed the impact of *H. pylori* eradication therapies on gut microbiota composition, only two studies focused on pediatric populations, one involving children aged 3 to 14 and the other adolescents aged 15 to 16.

Among the studies evaluating *H. pylori* eradication regimens supplemented with probiotics, a range of strains were employed, including *Clostridium butyricum* (MIYA-BM^®^), *Bacillus subtilis*, *Enterococcus faecium*, and *Saccharomyces boulardii*. These were administered in various forms, tablets, enteric capsules, and powders, at differing doses and durations. Despite this heterogeneity, most studies reported favorable outcomes such as reduced dysbiosis, improved gastrointestinal symptoms, and enhanced eradication rates. Only one study involving pediatric participants aged 7 to 8 years also observed symptom relief and microbial stabilization with probiotic use.

To complement the narrative description above, the following tables provide a structured summary of microbiome changes observed at Week 2 and during long-term follow-up. At Week 2, alpha diversity was reported to decrease in nearly half of the studies, while beta diversity showed significant compositional shifts in a subset of cases, reflecting the early impact of combined antibiotic and probiotic therapy (**Table 4**). Phylum-level changes at this time point highlighted marked reductions in *Firmicutes* and *Bacteroidetes*, alongside increased levels of *Proteobacteria* and *Fusobacteria*, with certain taxa found to be more abundant in the probiotic-treated groups (**Table 5**). At the genus level, notable changes included decreases in *Bacteroides*, *E. coli*, and *Enterococcus*, while beneficial genera such as *Bifidobacterium*, *Lactobacillus*, and *Clostridium butyricum* were more frequently preserved or increased in probiotic groups (**Table 6**). Long-term follow-up (1, 2, and 12 months) showed that most bacterial groups gradually returned to baseline, with transient fluctuations, such as temporary elevations in *Enterococcus* and *Bacillus*, resolving by Week 6 in almost all studies (**Table 7**).

**Table 4:** Microbiome diversity changes at week 2 following combined antibiotic and probiotic therapy.

Type	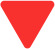 Decrease	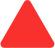 Increase	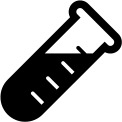 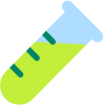 Significant change	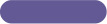 No change
Alpha	44.4%	—	—	—
Beta	—	11.1%	22.2%	—

This table summarizes the observed microbiome diversity changes at Week 2 in studies involving combined antibiotic and probiotic interventions. The percentages represent the proportion of studies reporting each type of change in diversity.

**Table 5:** Phylum-level gut microbiota changes at week 2 following antibiotic and probiotic therapy.

Phylum	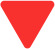 Increase	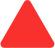 Decrease	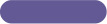 No Change	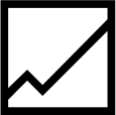 Higher in Probiotics Group
Bacteroidetes	—	44.4%	—	11.1%
Firmicutes	—	55.5%	—	—
Proteobacteria	44.4%	11.1%	—	—
Fusobacteria	—	22.2%	—	—
Actinobacteria	11.1%	11.1%	—	—

This table displays phylum-level microbiome changes observed at Week 2 following combined antibiotic and probiotic treatment. Percentages reflect the proportion of studies reporting each type of change in relative abundance for the specified phyla.

**Table 6:** Genus-level gut microbiota changes at week 2 following antibiotic and probiotic therapy.

Genus	▲ Increase	▼ Decrease	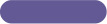 No change	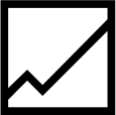 Higher in probiotics group
Bacteroides	—	11.1%	—	11.1%
E. coli	—	—	22.2%	Lower in probiotics group
Bifidobacterium	11.1% (gastric mucosa)	22.2%	No change in B. bifidum	11.1%
Lactobacillus	11.1% (gastric mucosa)	11.1%	No change in L. acidophilus	11.1%
Streptococcus	11.1%	—	—	—
Enterococcus	11.1%	—	—	Lower in probiotics group
Clostridium	—	—	—	11.1%
C. butyricum	11.1%	—	—	—

This table summarizes genus-level microbiome changes at Week 2 in response to combined antibiotic and probiotic treatment. The percentages represent the number of studies reporting each type of change in genus abundance.

**Table 7:** Long-term recovery of gut microbiota at 1, 2, and 12 months after antibiotic and probiotic therapy.

Group	✅ Restored to baseline	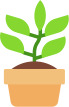 Temporary change (Week 4)	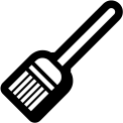 Resolved by week 6
All bacterial groups	Almost all studies	Enterococcus, Bacillus	Yes

This table summarizes findings on long-term microbiome recovery following antibiotic and probiotic intervention, assessed at 1, 2, and 12 months post-treatment.

100% of the studies that assessed different *H. pylori* eradication therapies in combination with probiotics reported that probiotic supplementation modulated gut microbiota composition in a treatment-specific manner. Across these studies, probiotics consistently attenuated the decline in alpha diversity and contributed to distinct beta diversity profiles between treatment groups. Several regimens, particularly those involving bismuth-based quadruple therapy, initially reduced beneficial taxa such as *Bifidobacterium* and *Lactobacillus*, while promoting opportunistic pathogens like *Klebsiella*, *Enterobacter*, and *Shigella*; however, the addition of probiotics helped suppress these shifts and facilitated microbial recovery. Notably, co-administration of *Clostridium butyricum* (CBM588) led to a dose-dependent decrease in *C. difficile* toxin A detection. Probiotic-treated groups also showed higher levels of beneficial genera (*Faecalibacterium*, *Bacteroides*) and more stable microbial communities, with several studies reporting near-complete restoration of baseline microbiota by week 22. Collectively, these findings underscore the consistent role of probiotics in mitigating dysbiosis and promoting microbiome resilience across all 9 studies evaluating combination therapies.

### 3.4 Findings of studies evaluating the gut microbiota alterations in *H. pylori* positive patients with gastric cancer

Five studies on microbiota changes in *H. pylori*-positive gastric cancer patients included participants aged 45 to 71, with one study focusing on patients under 18 (**Supplementary Table S5**). All studies used 16S rRNA gene sequencing for taxonomic analysis, while one also employed whole genome metagenomic analysis for a detailed view of microbial communities. Sixty percent of the studies utilized gastric biopsies, providing insights into bacterial populations in the stomach lining, while 40% analyzed stool samples. One study included both stool samples and gastric juice to enhance understanding of gut microbiota alterations in *H. pylori*-positive gastric cancer patients.

In terms of microbial findings, alpha diversity was reported to decrease in one study in stool samples and increase in two (both gastric biopsy). Beta diversity showed significant differences between *H. pylori*-positive and *H. pylori*-negative groups in one study involving stool samples. For specific bacterial groups, *Actinobacteria* levels decreased in two studies (gastric biopsy, gastric juice+stool) and increase in one (stool). *Bifidobacterium* levels also decreased in two studies (gastric biopsy, gastric juice+stool). Conversely, *Proteobacteria* levels in gastric juice and stool samples, as well as *Streptococcus* in gastric juice, biopsy, and stool samples, increased in two studies each. The *Bacteroides* genus showed mixed results, with an increase in one study (stool) and a decrease in another (gastric juice+stool). *Firmicutes* were dominant in early gastric cancer (EGC) in one study (gastric biopsy) but decreased in another (gastric juice+stool). *Haemophilus* was more prevalent in EGC compared to intestinal metaplasia (IM) in one gastric biopsy study, but its levels decreased in EGC in another biopsy study. Additionally, *Enterococcus* was uniquely found in EGC in one gastric biopsy study and increased in stool samples in another study.

## 4 Discussion

**Figure 1:**
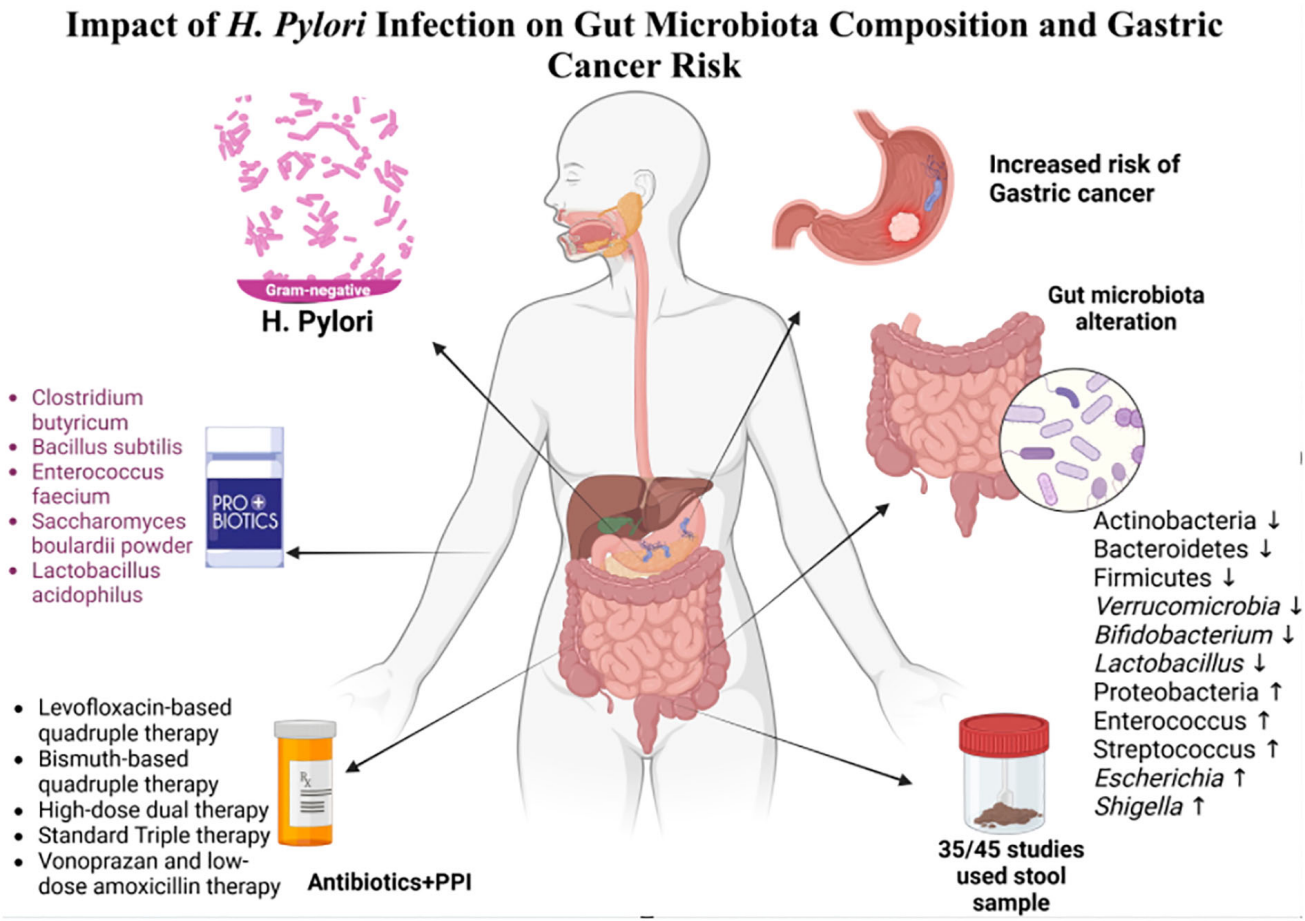
Impact of *H. pylori* infection on gut microbiota composition and gastric cancer risk.

This systematic review explores the relationship between gut microbiota composition and *H. pylori*, focusing on gastrointestinal changes with and without treatment. It also examines how *H. pylori* alter the gut microbiota in gastric cancer patients, highlighting its potential role in cancer progression (**Figure 1**).

As a component of the gastrointestinal ecosystem, *H. pylori* infection and its effect on gastric acid secretion can influence the GI microbiome and overall health of the host (Chen et al., 2021).

Results concerning the key measures of microbial richness and composition, alpha and beta diversity respectively (Miao et al., 2020), were mixed across all age groups. The mixed results concerning alpha diversity suggest that microbial richness may be sample-type dependent (Nearing et al., 2021). On the other hand, the findings of the beta diversity were much more consistent. Almost all studies reported that the beta diversity had distinguished between *Hp+* and *Hp-* individuals, with only one study reporting that the beta diversity remained unchanged. As mentioned in this study that a significance might be detected with a different analytical method or larger sample size (Dewayani et al., 2023).

On the phyla level, notable changes in the microbial community were observed in the young population. Gastric samples showed a decrease in the levels of *Actinobacteria, Firmicutes, and Bacteroidetes* but an increase in stool samples, highlighting a compartmentalized shift in microbial populations which suggests that *H. pylori* may potentially disrupt the gastric microbiota while also providing protection against external intestinal pathogens that lead to diarrhea (Kakiuchi et al., 2021; Martin-Nuñez et al., 2021). The observed decrease in gastric *Firmicutes* and *Bacteroidetes* could also be tied to changes in lipid and carbohydrate metabolism pathways, as these phyla play a crucial role in regulating lipid metabolism and maintaining energy homeostasis (Jian et al., 2022; Xiong, 2025). These metabolic and hormonal interactions may contribute to the unclear and controversial impact of *H. pylori* on growth and height in children as showed in 2 studies (Dharan and Wozny, 2022; Hong et al., 2022).

A consistent increase in *Proteobacteria*, a phylum often associated with inflammation and dysbiosis (Ibrahim and Belheouane, 2018), was observed across multiple studies in children and adolescents, indicating a potential link between *H. pylori* infection and the overgrowth of opportunistic pathogens (Sitkin et al., 2022). The increase in this phylum was also observed in adults; however, one study reported a decrease, indicating variability in the microbial response. This variability may be attributed to differences in ethnicity, genetic diversity, or diet (Dwiyanto et al., 2021).

In adults, *Bacteroidetes* showed a more consistent decrease, particularly in three studies. In contrast, *Verrucomicrobia*, a less common phylum, decreased in two studies, with a corresponding reduction in the genus *Akkermansia*, known for its role in maintaining gut mucosal integrity (Hajimohammad et al., 2024). This suggests that *H. pylori* infection may compromise the mucosal health of adults by depleting these beneficial bacteria. These findings underline an age-related microbial response to *H. pylori*, with greater consistency in adult studies regarding the depletion of certain beneficial bacterial groups (Walrath et al., 2021).

*H. pylori* infection elicits complex gut microbiota responses, differing by age. In children, probiotic genera like *Bifidobacterium* and *Lactobacillus* fluctuate, with some studies noting increases while others report declines. These bacteria support gut health by maturing the intestinal epithelium, maintaining its integrity, and regulating pH, alongside antimicrobial effects. Variations may be partly explained by regional hygiene practices (Gupta et al., 2020; Meliț et al., 2022). *Eubacterium*, a major butyrate producer, generally rises, particularly in children, benefiting gut health through anti-inflammatory short-chain fatty acid production (Du et al., 2024).

*Prevotella* levels consistently rise in infected individuals, suggesting that *H. pylori* promotes its carbohydrate-metabolizing capabilities across age groups (Prasoodanan et al., 2021). This elevation is consistent with other studies (Kakiuchi, 2023). Conversely, *Bacteroides* levels drop in adults, indicating disrupted gut balance (Zhang et al., 2023).

These shifts highlight a complex relationship between microbial changes and symptoms, with no single microbial group consistently predicting symptoms due to interspecies interactions (Simrén et al., 2012; Yu et al., 2023). Elevated levels of *Sphingomonas* in asymptomatic individuals suggest its potential as a distinguishing marker (Yu et al., 2023). This reduced levels of the bacterium in patients with persistent inflammation post-*H. pylori* eradication underscore its clinical relevance (Sung et al., 2020; Niu et al., 2021). Host immunity and environmental factors also shape microbial shifts (Woodhams et al., 2020). Adults show consistent microbial disruptions, particularly in beneficial bacteria, while children may tolerate *H. pylori* better, potentially activating protective responses against inflammation (Reyes, 2022; La Placa et al., 2025).

It has been shown that the colonization of the stomach and intestines by *H. pylori* leads to microbial alterations, and the treatment protocols used for its eradication also have a considerable impact on the microbiota in both the gastric and gut environments (Tohumcu et al., 2024).

Following *H. pylori* treatment without probiotic supplementation, alpha diversity generally declined, except in one study, likely due to variations in microbiome disruption caused by different treatments (Ramirez et al., 2020). In contrast, beta diversity findings were consistent, with nearly all studies reporting significant changes. At the phylum level, most studies observed a decrease in *Actinobacteria*, *Firmicutes*, and *Bacteroidetes* immediately after treatment (1–2 weeks). However, two studies found no change in *Firmicutes*, with *Actinobacteria* and *Bacteroidetes* levels even increasing, potentially due to treatment duration differences (7–14 days) (Lekang et al., 2022). *Proteobacteria* notably increased post-treatment, indicating gut dysbiosis (Gao et al., 2024).

At the genus level, *Lactobacilli*, *Bifidobacterium*, and *Prevotella* results varied, likely due to differing treatment regimens, while *Verrucomicrobia* consistently declined, suggesting compromised gut mucosal health (Fujio-Vejar et al., 2017; Mo et al., 2024). Most bacterial communities returned to baseline within 1–2 months, though some required 6–12 months. Findings suggest antibiotics rapidly decrease diversity and alter species abundance, leading to dysbiosis, with recovery often incomplete post-treatment, this underscores the importance of cautious antibiotic use to avoid lasting impacts on gut flora (Elvers et al., 2020).

When probiotics were co-administered during *H. pylori* eradication therapy, studies consistently reported beneficial outcomes across diverse probiotic regimens suggests a general protective effect of co-administration during *H. pylori* eradication therapy. These benefits likely stem from a combination of mechanisms, including modulation of host immune responses, suppression of opportunistic pathogens, and support for beneficial commensals (Raheem et al., 2021). However, substantial variability in the strains used, dosing schedules, and formulations complicates efforts to identify the most effective approach (Yang et al., 2024). Additionally, none of the studies employed head-to-head comparisons of different probiotic products, limiting the ability to draw conclusions about strain-specific efficacy. Standardization in future clinical protocols, including controlled comparisons of probiotic types and clearer reporting of microbiome outcomes, is essential to establish evidence-based recommendations for probiotic use in *H. pylori* treatment regimens (Wang et al., 2023).

Although current evidence supports the beneficial effects of probiotic supplementation during *H. pylori* eradication therapy, the lack of standardization across studies limits clinical applicability (Musazadeh et al., 2023). Nonetheless, several consistent findings allow for provisional recommendations. Strains such as *Lactobacillus rhamnosus GG, Saccharomyces boulardii, Clostridium butyricum* (particularly CBM588), and *Bifidobacterium lactis* have demonstrated efficacy in enhancing microbial recovery, reducing gastrointestinal side effects, and improving eradication rates (Imase et al., 2008). Most effective formulations delivered daily doses ranging from 1 × 10^9^ to 1 × 10^11^ CFU, with higher doses offering greater microbiota protection, especially in patients receiving bismuth-based quadruple therapy (Yao et al., 2023). Probiotic administration was most beneficial when initiated concurrently with antibiotic treatment and continued for at least 2 to 4 weeks post-therapy to support microbiome stabilization and reduce dysbiosis-related complications (Mullish et al., 2024). Enteric-coated capsules and multi-strain powder formulations were associated with improved gastrointestinal survival and colonization efficiency (Govaert et al., 2024). In light of these findings, probiotic co-administration, particularly with microbiota-disruptive regimens, should be considered a supportive adjunct to eradication therapy (di Vito et al., 2022). Future clinical trials should prioritize head-to-head comparisons of probiotic strains, define optimal dosing schedules, and assess long-term microbiota recovery to inform standardized probiotic protocols in *H. pylori* management.

At week 2 following antibiotic and probiotic intervention, a decrease in alpha diversity was observed in 44.4% of the studies. This reduction in microbial diversity underscores the early disruptions to the gut microbiome caused by antibiotic therapy, even with probiotic supplementation. This is likely because probiotics can be inhibited by antibiotics, leading to a temporary collapse in microbial diversity (Yang et al., 2023).

Significant shifts in beta diversity were noted in almost all studies. These changes reflect alterations in community composition and inter-individual variability post-treatment, suggesting that probiotics may promote divergent microbiome recovery pathways in certain individuals (Chandrasekaran et al., 2024).

Probiotic supplementation during treatment is associated with an enrichment of beneficial bacterial genera, including *Lactobacillus*, *Bifidobacterium*, *Bacteroidetes*, and *Clostridium*, while pathogenic bacteria such as *Enterococcus* are depleted (Stojanov et al., 2020; Yan and Polk, 2020; Sabit et al., 2023). These findings suggest that probiotics play a crucial role in restoring and maintaining a healthier gut microbiota, potentially mitigating antibiotic-induced disruptions (Dahiya and Nigam, 2023). Furthermore, multiple studies demonstrate that treatment regimens supplemented with probiotics yield higher *H. pylori* eradication rates compared to standard therapies, while also effectively reducing gastrointestinal side effects (Elghannam et al., 2024).

These findings underscore the significant impact that *H. pylori* eradication regimens can have on gut microbiota, especially in the absence of probiotic supplementation. The observed persistent dysbiosis following Bismuth-based Quadruple Therapy (BQT) aligns with prior studies demonstrating its broad-spectrum antimicrobial effect, which tends to disrupt not only *H. pylori* but also commensal bacteria essential for maintaining gut homeostasis (Hsu et al., 2018). The reduced alpha diversity and altered beta diversity, along with increased prevalence of opportunistic pathogens such as *Klebsiella pneumoniae* and *Enterococcus* spp., raise concerns about secondary infections and long-term gut health following BQT, especially in vulnerable populations (Zhou et al., 2020).

In contrast, the relatively minimal microbiota disruption seen with Vonoprazan–Amoxicillin dual therapy (VA-dual therapy) supports its growing reputation as a safer first-line treatment. The stability in both alpha and beta diversity metrics during and after therapy indicates a more targeted antimicrobial action and a lesser ecological disturbance to the gut environment (Peng et al., 2023).

Importantly, the consistent protective effect of probiotics across all nine studies reinforces their utility as adjunctive agents in *H. pylori* treatment protocols. Supplementation was associated with the preservation of beneficial genera such as *Bifidobacterium*, *Lactobacillus*, and *Faecalibacterium*, which are known to support gut barrier integrity and modulate immune responses (Yao et al., 2023). This effect not only enhanced microbiota recovery but also attenuated the bloom of opportunistic and potentially pathogenic taxa. These observations suggest that probiotic co-administration is particularly critical when using regimens known for high microbial impact, such as BQT (Yao et al., 2023).

Taken together, the data suggests a paradigm shift in *H. pylori* treatment strategy may be warranted, prioritizing regimens like VA-dual therapy for their microbiota-sparing properties, and integrating probiotics as a standard co-treatment to mitigate the collateral damage of antibiotics. Moreover, future research should explore personalized approaches that balance eradication efficacy with preservation of microbiome integrity (Keikha and Karbalaei, 2021).

While all included studies examined the impact of *H. pylori* eradication therapies, either with or without probiotic supplementation, on gut microbiota composition, only three specifically focused on pediatric populations: one involving children aged 3 to 14 years, another adolescents aged 15 to 16 years, and a third with participants aged 7 to 8 years. The remainder of the studies enrolled adult participants, thereby limiting the ability to draw definitive conclusions about age-related differences in post-treatment dysbiosis (Kang et al., 2025). The broad age range across studies (3 to 80 years) further highlights a significant limitation in assessing age-specific susceptibility to microbiota disruption induced by antimicrobials or probiotics (Kang et al., 2025). Evidence from the broader microbiome literature suggests that children may demonstrate greater microbial resilience and more rapid recovery due to developmental plasticity and distinct immune regulatory profiles (Schoultz et al., 2025). However, the underrepresentation of pediatric cohorts in the current dataset prevented a thorough investigation of these age-dependent effects (Mohammadkhah et al., 2018). Moreover, most studies failed to stratify outcomes by age or control for key confounding variables such as diet, immune status, comorbidities, and microbiome developmental stage, all of which are known to influence the risk and severity of dysbiosis (Vujkovic-Cvijin et al., 2020). These limitations underscore the need for future studies to explicitly consider age as a biological variable and to systematically evaluate its role in shaping microbiota responses to *H. pylori* eradication regimens. While this review acknowledges the limited availability of pediatric data, this gap warrants stronger emphasis given the potential for age-specific differences in microbiota composition, immune maturation, and treatment response (Budzinski et al., 2024). Without age-specific data, clinical recommendations risk being disproportionately informed by adult-centric findings, which may not generalize to younger cohorts (Budzinski et al., 2024). Therefore, there is an urgent need for future studies to systematically incorporate pediatric populations and conduct age-stratified analyses (Gschwendtner et al., 2019). Such efforts are essential not only to optimize eradication protocols for younger individuals but also to understand how early-life microbiota disruptions might influence long-term gastrointestinal and metabolic health (Gschwendtner et al., 2019).

The studies collectively highlight the pivotal role of the gut microbiome, particularly in *H. pylori*-positive patients, in the development and progression of gastric cancer. Several studies demonstrate notable shifts in microbial diversity and composition, closely associated with the severity of gastric conditions and the presence of cancer.

For instance, two studies report a significant decline in *Bifidobacterium* levels, especially in patients with severe gastric cancer. These bacteria play a key role in anti-inflammatory responses, and their depletion may contribute to worsening gastric health (Virk et al., 2024). Simultaneously, elevated levels of inflammation-associated genera like *Dialister* and *Prevotella* suggest a pro-inflammatory environment, potentially accelerating tissue damage and carcinogenesis (Smet et al., 2022; Anthamatten et al., 2024). Similarly, early gastric cancer patients exhibited increased levels of pathogenic bacteria, such as *Proteobacteria*, *Enterococcus*, *Streptococcus*, and *Escherichia-Shigella*, further fostering a pro-inflammatory state that could promote cancer progression (Wang et al., 2020).

Interestingly, a 2023 study from Japan observed a reduction in *Haemophilus* in early gastric cancer (EGC) patients who had undergone successful *H. pylori* eradication. In contrast, a 2021 study from Portugal found higher levels of *Haemophilus* in EGC patients. This discrepancy may be attributed to the successful eradication of *H. pylori* in the former study, leading to a significant shift in the gastric microbiota, while in the latter study, the presence of early-stage cancer without prior eradication therapy could explain the higher *Haemophilus* levels (Xu et al., 2020).

## 5 Limitations of the studies

This systematic review offers a comprehensive overview of gut microbiota diversity and alterations in patients with *H. pylori* infection. However, the study has several limitations.

Firstly, the lack of detailed dietary information across most included studies is a significant limitation, as diet is crucial in shaping gut microbiota composition and diversity. This absence may have influenced the reported outcomes, introducing confounding factors related to participants’ varying dietary habits.

Secondly, considerable heterogeneity exists among the studies, particularly regarding the timing of taxonomic analysis after *H. pylori* treatment cessation, which may affect result comparability since gut microbiota can fluctuate over time post-treatment. Beyond timing, this heterogeneity stems from multiple methodological and contextual variables. These include differences in sequencing techniques (e.g., 16S rRNA gene sequencing versus shotgun metagenomics), which affect taxonomic depth and functional profiling; variation in sample types (gastric biopsies, gastric fluid, or stool); and disparities in patient demographics, such as age, geography, diet, and disease stage. Additionally, treatment regimens varied widely in antibiotic combinations, durations, and probiotic use, while follow-up intervals were inconsistent across studies. The absence of standardized protocols and analytical approaches further compounds this variability. Together, these factors limit the comparability of findings and the strength of generalizable conclusions. Future research should aim to minimize heterogeneity through harmonized methodologies and incorporate stratified analyses to account for population and protocol differences.

Thirdly, there is a scarcity of studies focusing on pediatric populations, with only two examining the impact of *H. pylori* treatment on children and adolescents. This limits the generalizability of findings to younger age groups, as gut microbiota responses to treatment may differ due to developmental variations in the microbiome and immune system.

Fourth, most of the studies used 16S rRNA sequencing as the primary method for analysing the gut microbiota. Although this technique is commonly employed, it has limitations in terms of taxonomic resolution and does not provide detailed functional or species-level information as effectively as other methods like shotgun metagenomics (Muhamad Rizal et al., 2020). As a result, this approach may lead to an incomplete understanding of the role of the microbiome in the progression of gastric cancer.

Finally, many studies assessing *H. pylori* treatment effects on gut microbiota lacked control groups of *H. pylori*-negative healthy individuals or untreated *H. pylori*-positive individuals. The absence of these controls hampers the ability to accurately evaluate the specific effects of eradication therapy on gut microbial composition, making it challenging to attribute observed changes to treatment rather than natural variations in the gut microbiome or *H. pylori* infection progression. This significantly limits the strength of the conclusions drawn from these studies.

## 6 Conclusion

The systematic review indicates that *H. pylori* significantly modify gut microbiota, demonstrating distinct patterns between age groups. Adults experience consistent microbial disruptions, whereas children exhibit variability and potential protective benefits. Antibiotic treatment temporarily decreases microbial diversity, leading to an increase in pathogenic *Proteobacteria*. Probiotic administration facilitates the restoration of beneficial bacteria. In gastric cancer patients infected with *H. pylori*, microbial shifts suggest a possible role of dysbiosis in cancer progression, highlighting the necessity for further research to elucidate these interactions and enhance therapeutic strategies.

## Data Availability

The original contributions presented in the study are included in the article/**Supplementary Material**. Further inquiries can be directed to the corresponding author.

## References

[B1] Alexander S. M. Retnakumar R. J. Chouhan D. Devi T. N. B. Dharmaseelan S. Devadas K. . (2021). *Helicobacter pylori* in human stomach: the inconsistencies in clinical outcomes and the probable causes. Front. Microbiol. 12. doi: 10.3389/fmicb.2021.713955, PMID: 34484153 PMC8416104

[B2] Ali A. AlHussaini K. I. (2024). *Helicobacter pylori*: A contemporary perspective on pathogenesis, diagnosis and treatment strategies. Microorganisms. 12, 222. doi: 10.3390/microorganisms12010222, PMID: 38276207 PMC10818838

[B3] Anthamatten L. Rogalla von Bieberstein P. Menzi C. Zünd J. N. Lacroix C. de Wouters T. . (2024). Stratification of human gut microbiomes by succinotype is associated with inflammatory bowel disease status. Microbiome. 12, 186. doi: 10.1186/s40168-024-01897-8, PMID: 39350289 PMC11441152

[B4] Baj J. Forma A. Sitarz M. Portincasa P. Garruti G. Krasowska D. . (2020). *Helicobacter pylori* virulence factors—mechanisms of bacterial pathogenicity in the gastric microenvironment. Cells. 10, 27. doi: 10.3390/cells10010027, PMID: 33375694 PMC7824444

[B5] Bakhti S. Z. Latifi-Navid S. (2021). Interplay and cooperation of *Helicobacter pylori* and gut microbiota in gastric carcinogenesis. BMC Microbiol. 21, 258. doi: 10.1186/s12866-021-02315-x, PMID: 34556055 PMC8461988

[B6] Bornschein J. Pritchard D. M. (2022). Myths and misconceptions in the management of *Helicobacter pylori* infection. Frontline Gastroenterol. 13, 245–253. doi: 10.1136/flgastro-2021-101826, PMID: 35493626 PMC8996102

[B7] Budzinski L. Sempert T. Lietz L. Maier R. Kang G. U. von Stuckrad A. S. L. . (2024). Age-stratification reveals age-specific intestinal microbiota signatures in juvenile idiopathic arthritis. Mol. Cell Pediatr. 11, 12. doi: 10.1186/s40348-024-00186-6, PMID: 39653980 PMC11628465

[B8] Chandrasekaran P. Weiskirchen S. Weiskirchen R. (2024). Effects of probiotics on gut microbiota: An overview. Int. J. Mol. Sci. 25, 6022. doi: 10.3390/ijms25116022, PMID: 38892208 PMC11172883

[B9] Charitos I. A. D’Agostino D. Topi S. Bottalico L. (2021). 40 years of *Helicobacter pylori*: a revolution in biomedical thought. Gastroenterol. Insights 12, 111–135. doi: 10.3390/gastroent12020011

[B10] Chen C. C. Liou J. M. Lee Y. C. Hong T. C. El-Omar E. M. Wu M. S. (2021). The interplay between *Helicobacter pylori* and gastrointestinal microbiota. Gut Microbes 13, 1–22. doi: 10.1080/19490976.2021.1909459, PMID: 33938378 PMC8096336

[B11] Cunha E. S. Chen X. Sanz-Gaitero M. Mills D. J. Luecke H. (2021). Cryo-EM structure of *Helicobacter pylori* urease with an inhibitor in the active site at 2.0 Å resolution. Nat. Commun. 12, 230. doi: 10.1038/s41467-020-20485-6, PMID: 33431861 PMC7801526

[B12] Dahiya D. Nigam P. S. (2023). Antibiotic-therapy-induced gut dysbiosis affecting gut microbiota-brain axis and cognition: Restoration by intake of probiotics and synbiotics. Int. J. Mol. Sci. 24, 3074. doi: 10.3390/ijms24043074, PMID: 36834485 PMC9959899

[B13] De Cassai A. Boscolo A. Zarantonello F. Pettenuzzo T. Sella N. Geraldini F. . (2023). Enhancing study quality assessment: an in-depth review of risk of bias tools for meta-analysis—a comprehensive guide for anesthesiologists. J. Anesth. Analg Crit. Care 3, 44. doi: 10.1186/s44158-023-00129-z, PMID: 37932825 PMC10626791

[B14] Dewayani A. Afrida Fauzia K. Alfaray R. I. Waskito L. A. Doohan D. Rejeki P. S. . (2023). Gastric microbiome changes in relation with Helicobacter pylori resistance. PloS One 18, e0284958. doi: 10.1371/journal.pone.0284958, PMID: 37200323 PMC10194900

[B15] Dharan M. Wozny D. (2022). Helicobacter pylori infection and small intestinal bacterial overgrowth: more than what meets the eye. World J. Clin. Cases. 10, 7209–7214. doi: 10.12998/wjcc.v10.i21.7209, PMID: 36158005 PMC9353905

[B16] di Vito R. Conte C. Traina G. (2022). A multi-strain probiotic formulation improves intestinal barrier function by the modulation of tight and adherent junction proteins. Cells. 11, 2617. doi: 10.3390/cells11162617, PMID: 36010692 PMC9406415

[B17] Du Y. He C. An Y. Huang Y. Zhang H Fu W. . (2024). The role of short chain fatty acids in inflammation and body health. Int. J. Mol. Sci. 25, 7379. doi: 10.3390/ijms25137379, PMID: 39000498 PMC11242198

[B18] Duan Y. Xu Y. (2025). Dou, Y. et al. Helicobacter pylori and gastric cancer: mechanisms and new perspectives. J. Hematol. Oncol. 18, 10. doi: 10.1186/s13045-024-01654-2, PMID: 39849657 PMC11756206

[B19] Dwiyanto J. Hussain M. H. Reidpath D. Ong K. S. Qasim A. Lee S. W. H. . (2021). Ethnicity influences the gut microbiota of individuals sharing a geographical location: a cross-sectional study from a middle-income country. Sci. Rep. 11, 2618. doi: 10.1038/s41598-021-82311-3, PMID: 33514807 PMC7846579

[B20] Elghannam M. T. Hassanien M. H. Ameen Y. A. Turky E. A. Elattar G. M. ElRay A. A. . (2024). *Helicobacter pylori* and oral-gut microbiome: Clinical implications. Infection. 52, 289–300. doi: 10.1007/s15010-023-02115-7, PMID: 37917397 PMC10954935

[B21] Elvers K. T. Wilson V. J. Hammond A. Duncan L. Huntley A. L. Hay A. D. . (2020). Antibiotic-induced changes in the human gut microbiota for the most commonly prescribed antibiotics in primary care in the UK: a systematic review. BMJ Open 10. doi: 10.1136/bmjopen-2019-035677, PMID: 32958481 PMC7507860

[B22] Fong I. W. (2020). “ *Helicobacter pylori* infection: when should it be treated?,” in Current trends and concerns in infectious diseases. Emerging infectious diseases of the 21st century (Springer, Cham), 49–61. doi: 10.1007/978-3-030-36966-8_4

[B23] Frost F. Kacprowski T. Rühlemann M. Bang C. Franke A. Zimmermann K. . (2019). *Helicobacter pylori* infection associates with fecal microbiota composition and diversity. Sci. Rep. 9, 20100. doi: 10.1038/s41598-019-56631-4, PMID: 31882864 PMC6934578

[B24] Fujio-Vejar S. Vasquez Y. Morales P. Magne F. Vera-Wolf P. Ugalde J. A. . (2017). The gut microbiota of healthy Chilean subjects reveals a high abundance of the phylum verrucomicrobia. Front. Microbiol. 8. doi: 10.3389/fmicb.2017.01221, PMID: 28713349 PMC5491548

[B25] Gao W. Liu X. Zhang S. Wang J. Qiu B. Shao J. . (2024). Alterations in gut microbiota and inflammatory cytokines after administration of antibiotics in mice. Microbiol. Spectr. 12, e0309523. doi: 10.1128/spectrum.03095-23, PMID: 38899904 PMC11302321

[B26] Garman K. S. Brown H. Alagesan P. McCall S. J. Patierno S. Wang Q. . (2024). Helicobacter pylori testing prior to or at gastric cancer diagnosis and survival in a diverse US patient population. Gastric Cancer. 27, 28–35. doi: 10.1007/s10120-023-01448-4, PMID: 37985571 PMC10842898

[B27] Govaert M. Rotsaert C. Vannieuwenhuyse C. Duysburgh C. Medlin S. Marzorati M. . (2024). Survival of probiotic bacterial cells in the upper gastrointestinal tract and the effect of the surviving population on the colonic microbial community activity and composition. Nutrients. 16, 2791. doi: 10.3390/nu16162791, PMID: 39203927 PMC11357584

[B28] Gschwendtner S. Kang H. Thiering E. Kublik S. Fösel B. Schulz H. . (2019). Early life determinants induce sustainable changes in the gut microbiome of six-year-old children. Sci. Rep. 9, 12675. doi: 10.1038/s41598-019-49160-7, PMID: 31481742 PMC6722248

[B29] Gupta V. Kumar R. Sood U. Singhvi N. (2020). Reconciling hygiene and cleanliness: a new perspective from human microbiome. Indian J. Microbiol. 60, 37–44. doi: 10.1007/s12088-019-00839-5, PMID: 32089572 PMC7000587

[B30] Hajimohammad A. Fattahi E. Ardebili A. Kaboosi H. Ghaemi E. A. (2024). The frequency of *verrucomicrobia* in the intestinal mucus of patients with cancer and polyps compared to healthy individuals. Jundishapur J. Microbiol. 17, e146159. doi: 10.5812/jjm-146159

[B31] Hong T. Jiang X. Zou J. Yang J. Zhang H. Mai H. . (2022). Hepatoprotective effect of curcumin against bisphenol A-induced hepatic steatosis via modulating gut microbiota dysbiosis. J. Nutr. Biochem. 109, 109103. doi: 10.1016/j.jnutbio.2022.109103, PMID: 35780999

[B32] Hsu P. I. Pan C. Y. Kao J. Y. Tsay F. W. Peng N. J. Kao S. S. . (2018). Helicobacter pylori eradication with bismuth quadruple therapy leads to dysbiosis of gut microbiota with an increased relative abundance of Proteobacteria and decreased relative abundances of Bacteroidetes and Actinobacteria. Helicobacter. 23, e12498. doi: 10.1111/hel.12498, PMID: 29897654

[B33] Huang H. Zhong W. Wang X. Yang Y. Wu T. Chen R. . (2023). The role of gastric microecological dysbiosis in gastric carcinogenesis. Front. Microbiol. 14. doi: 10.3389/fmicb.2023.1218395, PMID: 37583514 PMC10423824

[B34] Ibrahim S. Belheouane M. (2018). “Methods for microbiota analysis: sample collection and laboratory methods,” in The Microbiome in Rheumatic Diseases and infection. Eds. Ragab G. Atkinson T. Stoll M. (Springer, Cham). doi: 10.1007/978-3-319-79026-8_2

[B35] Imase K. Takahashi M. Tanaka A. Tokunaga K. Sugano H. Tanaka M. . (2008). Efficacy of Clostridium butyricum preparation concomitantly with Helicobacter pylori eradication therapy in relation to changes in the intestinal microbiota. Microbiol. Immunol. 52, 156–161. doi: 10.1111/j.1348-0421.2008.00026.x, PMID: 18402597

[B36] Jian Z. Zeng L. Xu T. Sun S. Yan S. Zhao S. . (2022). The intestinal microbiome associated with lipid metabolism and obesity in humans and animals. J. Appl. Microbiol. 133, 2915–2930. doi: 10.1111/jam.15740, PMID: 35882518 PMC9804742

[B37] Kakiuchi T. (2023). Commentary: Association between Helicobacter pylori infection and metabolic syndrome. Front. Endocrinol. (Lausanne). 14. doi: 10.3389/fendo.2023.1270855, PMID: 37670882 PMC10475952

[B38] Kakiuchi T. Tanaka Y. Ohno H. Matsuo M. Fujimoto K. (2021). *Helicobacter pylori* infection-induced changes in the intestinal microbiota of 14-year-old or 15-year-old Japanese adolescents: a cross-sectional study. BMJ Open 11, e047941. doi: 10.1136/bmjopen-2020-047941, PMID: 34215607 PMC8256750

[B39] Kang D. W. Lee J. W. Park M. Y. Kim S. H. Um Y. H. Wang S. M. . (2025). Impact of Helicobacter pylori eradication on age-specific risk of incident dementia in patients with peptic ulcer disease: a nationwide population-based cohort study. Geroscience. 47, 1161–1174. doi: 10.1007/s11357-024-01284-z, PMID: 39129052 PMC11872846

[B40] Keikha M. Karbalaei M. (2021). Probiotics as the live microscopic fighters against *Helicobacter pylori* gastric infections. BMC Gastroenterol. 21, 388. doi: 10.1186/s12876-021-01977-1, PMID: 34670526 PMC8527827

[B41] La Placa G. Covino M. Candelli M. Gasbarrini A. Franceschi F. Merra G. (2025). Relationship between human microbiome and *helicobacter pylori* . Microbiol. Res. 16, 24. doi: 10.3390/microbiolres16010024

[B42] Lekang K. Shekhar S. Berild D. Petersen F. C. Winther-Larsen H. C. (2022). Effects of different amoxicillin treatment durations on microbiome diversity and composition in the gut. PloS One 17. doi: 10.1371/journal.pone.0275737, PMID: 36301847 PMC9612567

[B43] Martin-Nuñez G. M. Cornejo-Pareja I. Clemente-Postigo M. Tinahones F. J. (2021). Gut microbiota: the missing link between *helicobacter pylori* infection and metabolic disorders? Front. Endocrinol. (Lausanne) 12. doi: 10.3389/fendo.2021.639856, PMID: 34220702 PMC8247771

[B44] Meliț L. E. Mărginean C. O. Săsăran M. O. (2022). The challenges of eradicating pediatric *helicobacter pylori* infection in the era of probiotics. Children (Basel). 9, 795. doi: 10.3390/children9060795, PMID: 35740732 PMC9222169

[B45] Miao R. Wan C. Wang Z. (2020). The relationship of gastric microbiota and Helicobacter pylori infection in pediatrics population. Helicobacter. 25. doi: 10.1111/hel.12676, PMID: 31762120

[B46] Mo C. Lou X. Xue J. Shi Z. Zhao Y. Wang F. . (2024). The influence of *Akkermansia muciniphila* on intestinal barrier function. Gut Pathog. 16, 41. doi: 10.1186/s13099-024-00635-7, PMID: 39097746 PMC11297771

[B47] Mohammadkhah A. I. Simpson E. B. Patterson S. G. Ferguson J. F. (2018). Development of the gut microbiome in children, and lifetime implications for obesity and cardiometabolic disease. Children (Basel). 5, 160. doi: 10.3390/children5120160, PMID: 30486462 PMC6306821

[B48] Muhamad Rizal N. S. Neoh H.-m. Ramli R. Periyasamy P. R. A./K. Hanafiah A. Abdul Samat M. N. . (2020). Advantages and limitations of 16S rRNA next-generation sequencing for pathogen identification in the diagnostic microbiology laboratory: Perspectives from a middle-income country. Diagnostics (Basel). 10, 816. doi: 10.3390/diagnostics10100816, PMID: 33066371 PMC7602188

[B49] Mullish B. H. Michael D. R. Dabcheva M. Webberley T. S. Coates N. John D. A. . (2024). A double-blind, randomized, placebo-controlled study assessing the impact of probiotic supplementation on the symptoms of irritable bowel syndrome in females. Neurogastroenterol Motil. 36, e14751. doi: 10.1111/nmo.14751, PMID: 38287443

[B50] Musazadeh V. Nazari A. Faghfouri A. H. Emami M. Kavyani Z. Zokaei M. . (2023). The effectiveness of treatment with probiotics in *Helicobacter pylori* eradication: results from an umbrella meta-analysis on meta-analyses of randomized controlled trials. Food Funct. 14, 7654–7662. doi: 10.1039/d3fo00300k, PMID: 37540067

[B51] Nearing J. T. Comeau A. M. Langille M. G. I. (2021). Identifying biases and their potential solutions in human microbiome studies. Microbiome. 9, 113. doi: 10.1186/s40168-021-01059-0, PMID: 34006335 PMC8132403

[B52] Niu Z. Y. Li S. Z. Shi Y. Y. Xue Y. (2021). Effect of gastric microbiota on quadruple *Helicobacter pylori* eradication therapy containing bismuth. World J. Gastroenterol. 27, 3913–3924. doi: 10.3748/wjg.v27.i25.3913, PMID: 34321854 PMC8291010

[B53] Öztekin M. Yılmaz B. Ağagündüz D. Capasso R. (2021). Overview of *Helicobacter pylori* infection: clinical features, treatment, and nutritional aspects. Diseases. 9, 66. doi: 10.3390/diseases9040066, PMID: 34698140 PMC8544542

[B54] Peng Y. Lei X. Yang Q. Zhang G. He S. Wang M. . (2024). *Helicobacter pylori* CagA-mediated ether lipid biosynthesis promotes ferroptosis susceptibility in gastric cancer. Exp. Mol. Med. 56, 441–452. doi: 10.1038/s12276-024-01167-5, PMID: 38383581 PMC10907675

[B55] Peng R. Zhang Z. Qu Y. Chen W. (2023). The impact of *Helicobacter pylori* eradication with vonoprazan-amoxicillin dual therapy combined with probiotics on oral microbiota: a randomized double-blind placebo-controlled trial. Front. Microbiol. 14. doi: 10.3389/fmicb.2023.1273709, PMID: 37849923 PMC10577438

[B56] Prasoodanan P. K. Sharma A. K. Mahajan S. Dhakan D. B. Maji A. Scaria J. . (2021). Western and non-western gut microbiomes reveal new roles of Prevotella in carbohydrate metabolism and mouth-gut axis. NPJ Biofilms Microbiomes. 7, 77. doi: 10.1038/s41522-021-00248-x, PMID: 34620880 PMC8497558

[B57] Raheem A. Liang L. Zhang G. Cui S. (2021). Modulatory effects of probiotics during pathogenic infections with emphasis on immune regulation. Front. Immunol. 12. doi: 10.3389/fimmu.2021.616713, PMID: 33897683 PMC8060567

[B58] Ramirez J. Guarner F. Bustos Fernandez L. Maruy A. Sdepanian V. L. Cohen H. . (2020). Antibiotics as major disruptors of gut microbiota. Front. Cell Infect. Microbiol. 10. doi: 10.3389/fcimb.2020.572912, PMID: 33330122 PMC7732679

[B59] Reyes V. E. (2022). *Helicobacter pylori* immune response in children versus adults. Med. Res. Arch. 10, 3370. doi: 10.18103/mra.v10i12.3370, PMID: 37936946 PMC10629867

[B60] Sabit H. Kassab A. Alaa D. Mohamed S. Abdel-Ghany S. Mansy M. . (2023). The effect of probiotic supplementation on the gut-brain axis in psychiatric patients. Curr. Issues Mol. Biol. 45, 4080–4099. doi: 10.3390/cimb45050260, PMID: 37232729 PMC10217264

[B61] Schoultz I. Claesson M. J. Dominguez-Bello M. G. Fåk Hållenius F. Konturek P. Korpela K. . (2025). Gut microbiota development across the lifespan: Disease links and health-promoting interventions. J. Intern. Med. doi: 10.1111/joim.20089, PMID: 40270478 PMC12087861

[B62] Simrén M. Barbara G. Flint H. J. Spiegel B. M. R. Spiller R. C. Vanner S. . (2012). Intestinal microbiota in functional bowel disorders: a Rome foundation report. Gut. 62, 159–176. doi: 10.1136/gutjnl-2012-302167, PMID: 22730468 PMC3551212

[B63] Sitkin S. Lazebnik L. Avalueva E. Kononova S. Vakhitov T. . (2022). Gastrointestinal microbiome and Helicobacter pylori: eradicate, leave it as it is, or take a personalized approach? World J. Gastroenterol. 28, 766–774. doi: 10.3748/wjg.v28.i7.766, PMID: 35317277 PMC8891730

[B64] Slim K. Nini E. Forestier D. Kwiatkowski F. Panis Y. Chipponi J. (2003). Methodological index for non-randomized studies (MINORS): development and validation of a new instrument. ANZ J. Surg. 73, 712–716. doi: 10.1046/j.1445-2197.2003.02748.x, PMID: 12956787

[B65] Smet A. Kupcinskas J. Link A. Hold G. L. Bornschein J. (2022). The role of microbiota in gastrointestinal cancer and cancer treatment: chance or curse? Cell Mol. Gastroenterol. Hepatol. 13, 857–874. doi: 10.1016/j.jcmgh.2021.08.013, PMID: 34506954 PMC8803618

[B66] Stojanov S. Berlec A. Štrukelj B. (2020). The influence of probiotics on the firmicutes/bacteroidetes ratio in the treatment of obesity and inflammatory bowel disease. Microorganisms. 8, 1715. doi: 10.3390/microorganisms8111715, PMID: 33139627 PMC7692443

[B67] Sung J. Coker O. O. Chu E. Szeto C. H. Luk S. S. Lau H. C. H. . (2020). Gastric microbes associated with gastric inflammation, atrophy, and intestinal metaplasia 1 year after *Helicobacter pylori* eradication. Gut. 69, 1572–1580. doi: 10.1136/gutjnl-2019-319826, PMID: 31974133 PMC7456733

[B68] Tohumcu E. Kaitsas F. Bricca L. Ruggeri A. Gasbarrini A. Cammarota G. . (2024). *Helicobacter pylori* and the human gastrointestinal microbiota: A multifaceted relationship. Antibiotics (Basel). 13, 584. doi: 10.3390/antibiotics13070584, PMID: 39061266 PMC11274338

[B69] Virk M. S. Virk M. A. He Y. Tufail T. Gul M. Qayum A. . (2024). The anti-inflammatory and curative exponent of probiotics: A comprehensive and authentic ingredient for the sustained functioning of major human organs. Nutrients. 16, 546. doi: 10.3390/nu16040546, PMID: 38398870 PMC10893534

[B70] Vujkovic-Cvijin I. Sklar J. Jiang L. Natarajan L. Knight R. Belkaid Y. (2020). Host variables confound gut microbiota studies of human disease. Nature. 587, 448–454. doi: 10.1038/s41586-020-2881-9, PMID: 33149306 PMC7677204

[B71] Walrath T. Dyamenahalli K. U. Hulsebus H. J. McCullough R. L. Idrovo J.-P. Boe D. M. . (2021). Age-related changes in intestinal immunity and the microbiome. J. Leukoc. Biol. 109, 1045–1061. doi: 10.1002/JLB.3RI0620-405RR, PMID: 33020981 PMC8139861

[B72] Wang J. Chen W. D. Wang Y. D. (2020). The relationship between gut microbiota and inflammatory diseases: The role of macrophages. Front. Microbiol. 11. doi: 10.3389/fmicb.2020.01065, PMID: 32582063 PMC7296120

[B73] Wang Y. Wang X. Cao X. Y. Zhu H. L. Miao L. (2023). Comparative effectiveness of different probiotics supplements for triple helicobacter pylori eradication: a network meta-analysis. Front. Cell Infect. Microbiol. 13. doi: 10.3389/fcimb.2023.1120789, PMID: 37256113 PMC10226649

[B74] Woodhams D. C. Bletz M. C. Becker C. G. Bender H. A. Buitrago Rosas D. Diebboll H. . (2020). Host-associated microbiomes are predicted by immune system complexity and climate. Genome Biol. 21, 23. doi: 10.1186/s13059-020-01955-y, PMID: 32014020 PMC6996194

[B75] Xiong S. (2025). Gut-microbiota-driven lipid metabolism: mechanisms and applications in swine production. Metabolites. 15, 248. doi: 10.3390/metabo15040248, PMID: 40278377 PMC12029090

[B76] Xu L. Surathu A. Raplee I. Chockalingam A. Stewart S. Walker L. . (2020). The effect of antibiotics on the gut microbiome: A metagenomics analysis of microbial shift and gut antibiotic resistance in antibiotic treated mice. BMC Genomics 21, 263. doi: 10.1186/s12864-020-6665-2, PMID: 32228448 PMC7106814

[B77] Yadav M. Chauhan N. S. (2024). Role of gut-microbiota in disease severity and clinical outcomes. Brief Funct. Genomics 23, 24–37. doi: 10.1093/bfgp/elac037, PMID: 36281758

[B78] Yan F. Polk D. B. (2020). Probiotics and probiotic-derived functional factors—mechanistic insights into applications for intestinal homeostasis. Front. Immunol. 11. doi: 10.3389/fimmu.2020.01428, PMID: 32719681 PMC7348054

[B79] Yang Z. Zhou Y. Han Z. He K. Zhang Y. Wu D. . (2024). The effects of probiotics supplementation on Helicobacter pylori standard treatment: an umbrella review of systematic reviews with meta-analyses. Sci. Rep. 14, 10069. doi: 10.1038/s41598-024-59399-4, PMID: 38697990 PMC11066092

[B80] Yang L. Zhu X. Zhao W. Wang J . (2023). Effect of combined probiotics on the intestinal ecosystem during triple therapy for eradication of *Helicobacter pylori* . J. Biol. Regul. Homeost Agents. 37, 1075–1080. doi: 10.23812/j.biol.regul.homeost.agents.20233702.109

[B81] Yao G. Fan X. Lu D. (2023). Efficacy and safety of probiotic-supplemented bismuth quadruple therapy for the treatment of *Helicobacter pylori* infection: a systematic review and meta-analysis. J. Int. Med. Res. 51, 3000605231203841. doi: 10.1177/03000605231203841, PMID: 37848344 PMC10586011

[B82] Yu T. Lu T. Deng W. Yao D. He C. Luo P. . (2023). Microbiome and function alterations in the gastric mucosa of asymptomatic patients with Helicobacter pylori infection. Helicobacter. doi: 10.1111/hel.12965, PMID: 36890119

[B83] Yu D. Meng X. de Vos W. M. Wu H. Fang X. Maiti A. K. (2021). Implications of gut microbiota in complex human diseases. Int. J. Mol. Sci. 22, 12661. doi: 10.3390/ijms222312661, PMID: 34884466 PMC8657718

[B84] Zhang L. Zhao M. Fu X. (2023). Gastric microbiota dysbiosis and Helicobacter pylori infection. Front. Microbiol. 14. doi: 10.3389/fmicb.2023.1153269, PMID: 37065152 PMC10098173

[B85] Zhou Y. Ye Z. Lu J. Miao S. Lu X. Sun H. . (2020). Long-term changes in the gut microbiota after 14-day bismuth quadruple therapy in penicillin-allergic children. Helicobacter. 25, e12721. doi: 10.1111/hel.12721, PMID: 32656891

