# Supplementary material for: The impact of Helicobacter pylori infection and eradication therapies on gut microbiota: a systematic review of microbial dysbiosis and its implications in gastric carcinogenesis
Source: Front Cell Infect Microbiol. 2025 Jul 7;15:1592977. doi: 10.3389/fcimb.2025.1592977 (PMC12277284; doi:10.3389/fcimb.2025.1592977)
Supplement: Supplementary file 1 [file Table1.docx]

- **Queries**: The search strings for PubMed:

Query of aim 1:

((("Gastrointestinal Microbiome"[Title/Abstract] OR "Microbiome, Gastrointestinal"[Title/Abstract] OR "Gastrointestinal Microbial Community"[Title/Abstract] OR "Gastrointestinal Microbial Communities"[Title/Abstract] OR "Microbial Community, Gastrointestinal"[Title/Abstract] OR "Gut Microbiome"[Title/Abstract] OR "Gut Microbiomes"[Title/Abstract] OR "Microbiome, Gut"[Title/Abstract] OR "Gut Microflora"[Title/Abstract] OR "Microflora, Gut"[Title/Abstract] OR "Gastrointestinal Microflora"[Title/Abstract] OR "Microflora, Gastrointestinal"[Title/Abstract] OR "Gastrointestinal Flora"[Title/Abstract] OR "Flora, Gastrointestinal"[Title/Abstract] OR "Gut Flora"[Title/Abstract] OR "Flora, Gut"[Title/Abstract] OR "Gastrointestinal Microbiota"[Title/Abstract] OR "Gastrointestinal Microbiotas"[Title/Abstract] OR "Microbiota, Gastrointestinal"[Title/Abstract] OR "Gut Microbiota"[Title/Abstract] OR "Gut Microbiotas"[Title/Abstract] OR "Microbiota, Gut"[Title/Abstract] OR "Intestinal Microbiome"[Title/Abstract] OR "Intestinal Microbiomes"[Title/Abstract] OR "Microbiome, Intestinal"[Title/Abstract] OR "Intestinal Flora"[Title/Abstract] OR "Flora, Intestinal"[Title/Abstract] OR "Intestinal Microbiota"[Title/Abstract] OR "Intestinal Microbiotas"[Title/Abstract] OR "Microbiota, Intestinal"[Title/Abstract] OR "Intestinal Microflora"[Title/Abstract] OR "Microflora, Intestinal"[Title/Abstract] OR "Enteric Bacteria"[Title/Abstract] OR "Bacteria, Enteric"[Title/Abstract] OR "Gastric Microbiome"[Title/Abstract] OR "Gastric Microbiomes"[Title/Abstract] OR "Microbiome, Gastric"[Title/Abstract]) AND ("Campylobacter pylori subsp. pylori"[Title/Abstract] OR "Campylobacter pyloridis"[Title/Abstract] OR "Campylobacter pylori"[Title/Abstract] OR "Helicobacter nemestrinae"[Title/Abstract] OR "Helicobacter pylori"[Title/Abstract])) NOT (Review[Publication Type])) NOT (systematic review[Publication Type])

Query of aim 2:

((("Gastrointestinal Microbiome"[Title/Abstract] OR "Microbiome, Gastrointestinal"[Title/Abstract] OR "Gastrointestinal Microbial Community"[Title/Abstract] OR "Gastrointestinal Microbial Communities"[Title/Abstract] OR "Microbial Community, Gastrointestinal"[Title/Abstract] OR "Gut Microbiome"[Title/Abstract] OR "Gut Microbiomes"[Title/Abstract] OR "Microbiome, Gut"[Title/Abstract] OR "Gut Microflora"[Title/Abstract] OR "Microflora, Gut"[Title/Abstract] OR "Gastrointestinal Microflora"[Title/Abstract] OR "Microflora, Gastrointestinal"[Title/Abstract] OR "Gastrointestinal Flora"[Title/Abstract] OR "Flora, Gastrointestinal"[Title/Abstract] OR "Gut Flora"[Title/Abstract] OR "Flora, Gut"[Title/Abstract] OR "Gastrointestinal Microbiota"[Title/Abstract] OR "Gastrointestinal Microbiotas"[Title/Abstract] OR "Microbiota, Gastrointestinal"[Title/Abstract] OR "Gut Microbiota"[Title/Abstract] OR "Gut Microbiotas"[Title/Abstract] OR "Microbiota, Gut"[Title/Abstract] OR "Intestinal Microbiome"[Title/Abstract] OR "Intestinal Microbiomes"[Title/Abstract] OR "Microbiome, Intestinal"[Title/Abstract] OR "Intestinal Flora"[Title/Abstract] OR "Flora, Intestinal"[Title/Abstract] OR "Intestinal Microbiota"[Title/Abstract] OR "Intestinal Microbiotas"[Title/Abstract] OR "Microbiota, Intestinal"[Title/Abstract] OR "Intestinal Microflora"[Title/Abstract] OR "Microflora, Intestinal"[Title/Abstract] OR "Enteric Bacteria"[Title/Abstract] OR "Bacteria, Enteric"[Title/Abstract] OR "Gastric Microbiome"[Title/Abstract] OR "Gastric Microbiomes"[Title/Abstract] OR "Microbiome, Gastric"[Title/Abstract]) AND ("Campylobacter pylori subsp. pylori"[Title/Abstract] OR "Campylobacter pyloridis"[Title/Abstract] OR "Campylobacter pylori"[Title/Abstract] OR "Helicobacter nemestrinae"[Title/Abstract] OR "Helicobacter pylori"[Title/Abstract]) AND ("Neoplasm, Stomach"[Title/Abstract] OR "Stomach Neoplasm"[Title/Abstract] OR "Gastric Neoplasms"[Title/Abstract] OR "Gastric Neoplasm"[Title/Abstract] OR "Neoplasm, Gastric"[Title/Abstract] OR "Neoplasms, Gastric"[Title/Abstract] OR "Neoplasms, Stomach"[Title/Abstract] OR "Cancer of Stomach"[Title/Abstract] OR "Stomach Cancers"[Title/Abstract] OR "Cancer of the Stomach"[Title/Abstract] OR "Gastric Cancer"[Title/Abstract] OR "Cancer, Gastric"[Title/Abstract] OR "Cancers, Gastric"[Title/Abstract] OR "Gastric Cancers"[Title/Abstract] OR "Stomach Cancer"[Title/Abstract] OR "Cancers, Stomach"[Title/Abstract] OR "Cancer, Stomach"[Title/Abstract] OR "Gastric Cancer, Familial Diffuse"[Title/Abstract])) NOT (review[Publication Type])) NOT (systematic review[Publication Type])

**The search strings for Scopus and Web of Science (searched in Title or Abstract) :**

Query of Aim 1:

("Gastrointestinal Microbiome" OR "Microbiome, Gastrointestinal" OR "Gastrointestinal Microbial Community" OR "Gastrointestinal Microbial Communities" OR "Microbial Community, Gastrointestinal" OR "Gut Microbiome" OR "Gut Microbiomes" OR "Microbiome, Gut" OR "Gut Microflora" OR "Microflora, Gut" OR "Gastrointestinal Microflora" OR "Microflora, Gastrointestinal" OR "Gastrointestinal Flora" OR "Flora, Gastrointestinal" OR "Gut Flora" OR "Flora, Gut" OR "Gastrointestinal Microbiota" OR "Gastrointestinal Microbiotas" OR "Microbiota, Gastrointestinal" OR "Gut Microbiota" OR "Gut Microbiotas" OR "Microbiota, Gut" OR "Intestinal Microbiome" OR "Intestinal Microbiomes" OR "Microbiome, Intestinal" OR "Intestinal Flora" OR "Flora, Intestinal" OR "Intestinal Microbiota" OR "Intestinal Microbiotas" OR "Microbiota, Intestinal" OR "Intestinal Microflora" OR "Microflora, Intestinal" OR "Enteric Bacteria" OR "Bacteria, Enteric" OR "Gastric Microbiome" OR "Gastric Microbiomes" OR "Microbiome, Gastric") AND ("Campylobacter pylori subsp. pylori" OR "Campylobacter pyloridis" OR "Campylobacter pylori" OR "Helicobacter nemestrinae" OR "Helicobacter pylori")

Query of Aim 2:

("Gastrointestinal Microbiome" OR "Microbiome, Gastrointestinal" OR "Gastrointestinal Microbial Community" OR "Gastrointestinal Microbial Communities" OR "Microbial Community, Gastrointestinal" OR "Gut Microbiome" OR "Gut Microbiomes" OR "Microbiome, Gut" OR "Gut Microflora" OR "Microflora, Gut" OR "Gastrointestinal Microflora" OR "Microflora, Gastrointestinal" OR "Gastrointestinal Flora" OR "Flora, Gastrointestinal" OR "Gut Flora" OR "Flora, Gut" OR "Gastrointestinal Microbiota" OR "Gastrointestinal Microbiotas" OR "Microbiota, Gastrointestinal" OR "Gut Microbiota" OR "Gut Microbiotas" OR "Microbiota, Gut" OR "Intestinal Microbiome" OR "Intestinal Microbiomes" OR "Microbiome, Intestinal" OR "Intestinal Flora" OR "Flora, Intestinal" OR "Intestinal Microbiota" OR "Intestinal Microbiotas" OR "Microbiota, Intestinal" OR "Intestinal Microflora" OR "Microflora, Intestinal" OR "Enteric Bacteria" OR "Bacteria, Enteric" OR "Gastric Microbiome" OR "Gastric Microbiomes" OR "Microbiome, Gastric") AND ("Campylobacter pylori subsp. pylori" OR "Campylobacter pyloridis" OR "Campylobacter pylori" OR "Helicobacter nemestrinae" OR "Helicobacter pylori") AND ("Neoplasm, Stomach" OR "Stomach Neoplasm" OR "Gastric Neoplasms" OR "Gastric Neoplasm" OR "Neoplasm, Gastric" OR "Neoplasms, Gastric" OR "Neoplasms, Stomach" OR "Cancer of Stomach" OR "Stomach Cancers" OR "Cancer of the Stomach" OR "Gastric Cancer" OR "Cancer, Gastric" OR "Cancers, Gastric" OR "Gastric Cancers" OR "Stomach Cancer" OR "Cancers, Stomach" OR "Cancer, Stomach" OR "Gastric Cancer, Familial Diffuse")

**Total number of articles for Query1:** 1,640

**Total number of articles after removing the duplicates for Query 1:** 808

**Total number of articles for Query2:** 371

**Total number of articles after removing the duplicates for Query2:** 208

Duplicates were removed with the help of AI.
