# Supplementary material for: The impact of Helicobacter pylori infection and eradication therapies on gut microbiota: a systematic review of microbial dysbiosis and its implications in gastric carcinogenesis
Source: Front Cell Infect Microbiol. 2025 Jul 7;15:1592977. doi: 10.3389/fcimb.2025.1592977 (PMC12277284; doi:10.3389/fcimb.2025.1592977)
Supplement: Supplementary file 3 [file Table3.docx]

**Query 1**

**Full-Text articles assessed for eligibility**

**(n=41)**

**Title-Abstract articles assessed for eligibility**

**(n=501)**

**Articles screened**

**(n=808)**

**Articles Identified through**

**databases searching (PubMed, Web of Science, Scopus) (n=1,640)**

**Articles after duplicates removed (n=808)**

**Identification**

**Articles Excluded**

**Reasons: Reviews, Animal studies**

**(n=307)**

**Screening**

**Title-Abstract**

**Articles Excluded for being out of topic**

**(n=460)**

**Eligibility**

**Full-Text articles excluded because of recent antibiotic use**

**(n=1)**

**Included**

**Studies included in the systematic review**

**(n=40)**

**Query 2**

**Full-Text articles assessed for eligibility**

**(n=7)**

**Title-Abstract articles assessed for eligibility**

**(n=16)**

**Articles screened**

**(n=208)**

**Articles Identified through**

**databases searching (PubMed, Web of Science, Scopus) (n=371)**

**Articles after duplicates removed (n=208)**

**Identification**

**Screening**

**Eligibility**

**Included**

**Full-Text articles excluded for tackling gastric diseases without gastric cancer**

**(n=2)**

**Articles included in the systematic review**

**(n=5)**

**Title-Abstract Articles Excluded**

**For being out of topic**

**(n=9)**

**Articles Excluded**

**Reasons: Reviews, Animal studies**

**(n=192)**
